# Pilot multicenter study to determine the utility of point-of-care ultrasound to predict difficulty of tracheal intubation using videolaryngoscopy with the McGrath™ Mac videolaryngoscope

**DOI:** 10.3389/fmed.2024.1406676

**Published:** 2024-07-19

**Authors:** Miguel A. Fernández-Vaquero, Nekari De Luis-Cabezón, Miguel A. García-Aroca, Jose M. Álvarez-Avello, Marc Vives-Santacana, Robert Greif, Eugenio D. Martinez-Hurtado, Diana Ly-Liu

**Affiliations:** ^1^Department of Anesthesiology, Clínica Universidad de Navarra, Madrid, Spain; ^2^School of Medicine, Navarra University, Navarra, Spain; ^3^Department of Anesthesiology, Basurto University Hospital, Bilbao, Spain; ^4^Department of Anesthesiology, Clínica Universidad de Navarra, Pamplona, Spain; ^5^School of Medicine, University of Bern, Bern, Switzerland; ^6^School of Medicine, Sigmund Freud University Vienna, Vienna, Austria; ^7^Department of Anesthesiology, Infanta Leonor University Hospital, Madrid, Spain

**Keywords:** airway management, tracheal intubation, videolaryngoscopy, video-assisted techniques, ultrasonography

## Abstract

**Background:**

Clinical airway screening tests used to predict difficulties during airway management have low sensitivity and specificity. Point-of-care airway ultrasound has described measurements related to problems with difficult direct laryngoscopy. Nevertheless, the correlation between ultrasound parameters and videolaryngoscopy has not been published yet. The aim of this multicenter, prospective observational pilot study was to evaluate the applicability of clinical parameters and ultrasound measurements to find potential tracheal intubation difficulties when videolaryngoscopy is used.

**Methods:**

Preoperatively, six clinical airway assessments were performed: (1) modified Mallampati score, (2) thyromental distance, (3) sternomental distance, (4) interincisal distance, (5) upper lip bite test, and (6) neck circumference. Six ultrasound parameters were measured in awake patients: (1) distance from skin to hyoid bone, (2) distance from skin to epiglottis, (3) hyomental distance in neutral head position, (4) hyomental distance in head-extended position, (5) distance from skin to the deepest part of the palate, and (6) sagittal tongue area. And finally, there was one ultrasound measure obtained in anesthetized patients, the compressed sagittal tongue area during videolaryngoscopy. The difficulty for tracheal intubation using a McGrath™ Mac videolaryngoscope, the percentage of glottic opening, and Cormack-Lehane grade were also assessed.

**Results:**

In this cohort of 119 subjects, tongue dimensions, particularly the sagittal tongue area, showed a robust association with increased intubation difficulty using videolaryngoscopy. A multiparametric model combining the following three ultrasound variables in awake patients: (a) the distance from skin to epiglottis, (b) the distance from skin to the deepest part of the palate, and (c) the sagittal tongue area, yielded a sensitivity of 92.3%, specificity of 94.5%, positive predictive value of 82.8%, and negative predictive value of 97.8% (*p* < 0.001).

**Conclusion:**

Point-of-care airway ultrasound emerges as a more useful tool compared to traditional clinical scales to anticipate possible challenges during videolaryngoscopic intubation.

## Introduction

The NAP4 report on complications associated with airway management in the UK, highlighted that airway physical examination did not adequately identify difficulties with airway management (1). A Cochrane meta-analysis (2) and a systematic review (3), revealed that there were no reliable clinical screening tests to predict difficult direct laryngoscopy or difficult tracheal intubation.

Ultrasonography, or insonation, has emerged as a crucial addition to bedside physical examination, along with inspection, palpation, percussion, and auscultation (4). For airway assessment, Point-Of-Care Ultrasound (POCUS) has gained importance in routine clinical practice for its ability to address focused questions, narrow differential diagnoses, and guide procedures (5), all with a short learning curve (6). At the same time, videolaryngoscopy has garnered attention for its benefits demonstrated in various investigations and meta-analyses (7, 8). Anesthesiologists are increasingly embracing both videolaryngoscopy and ultrasonography to optimize patient care.

Recent systematic reviews have established correlations between ultrasound measurements and difficulties during direct laryngoscopy performance and tracheal intubation (9–12). Nonetheless, there is a lack of knowledge about sonography and tracheal intubation using videolaryngoscopy. In contrast, clinical parameters associated with difficult intubation using videolaryngoscopy, such as a thick neck, male sex, macroglossia, diminished thyromental or sternothyroid distance, and previous elevated Cormack-Lehane grade, are well-documented by the Canadian Airway Focus Group (13).

The primary objective of the present study was to determine the effectiveness of clinical parameters and ultrasound measurements to assess the difficulty of tracheal intubation using videolaryngoscopy with a McGrath™ Mac videolaryngoscope.

The secondary objective was to stablish the relationship between the Percentage of Glottis Opening (POGO) score and the difficulty of tracheal intubation using videolaryngoscopy.

## Materials and methods

### Study design and participants

Ethical approval for this prospective cross-sectional, multicenter, observational pilot study was obtained from the Ethical Committees of Navarra University Hospital (Pamplona, Spain) and Euskadi (Vitoria, Spain). Approval was granted by María del Carmen Berasain Lasarte, Chairperson of the Ethical Committee of Navarra University Hospital, in February 2023 (Project ID: 2022.193), and by Arantza Hernández Gil, Chairperson of the Ethical Committee of Euskadi, in March 2023. The study was registered at clinicaltrials.gov (NCT05767099). All study participants signed written informed consent before their enrolment. The study adhered to the principles outlined in the Helsinki Declaration, followed the Good Clinical Practice guidelines, and complied with the Spanish legislation governing biomedical research. This study followed the Strengthening the Reporting of Observational Studies in Epidemiology (STROBE) guideline for cohort studies to ensure comprehensive and transparent reporting of observational research (14).

All adult participants aged 18–90 years undergoing elective surgical procedures which required general anesthesia were consecutively enrolled in participating centers. The recruitment time was of 5 months, from March to July 2023. Inclusion criteria consisted of an “American Society of Anesthesiologists” (ASA) physical status classification of 1 to 3. Exclusion criteria were body mass index (BMI) >35 kg/m^2^, pregnancy, cervical tumors or goiter, history of cervical radiation therapy, maxillofacial or cervical abnormalities, and inability or unwillingness to sign the informed consent.

Follow-up assessments were conducted the day of surgery, using hospital and medical records. The authors affirm the meticulousness of data collection and recording in the specifically designed data collection notebook. Additionally, the authors assure adherence to the study protocol throughout the trial, ensuring consistency and reliability of the collected data.

### Data collection, clinical parameters, and ultrasound measurements

During the preanesthetic evaluation, patient characteristics including age, sex, weight, height, and ASA physical status were recorded, along with six clinical airway screening tests: modified Mallampati score (MMS), thyromental distance (TMD), sternomental distance (SMD), interincisal distance (IID), upper lip bite test (ULBT), and neck circumference (NC) (2, 3).

Ultrasound measurements were taken in the operating theater with a high frequency linear probe (6–12 MHz) and a low frequency convex probe (1–6 MHz) (General Electric Logiq V2, GE Medical Systems, Jiangsu, China or Sonosite, Edge II, United States). The recommended guidelines for probe placement and penetration depth were followed (6).

### Pre-induction ultrasound measurements

**Figure 1:**
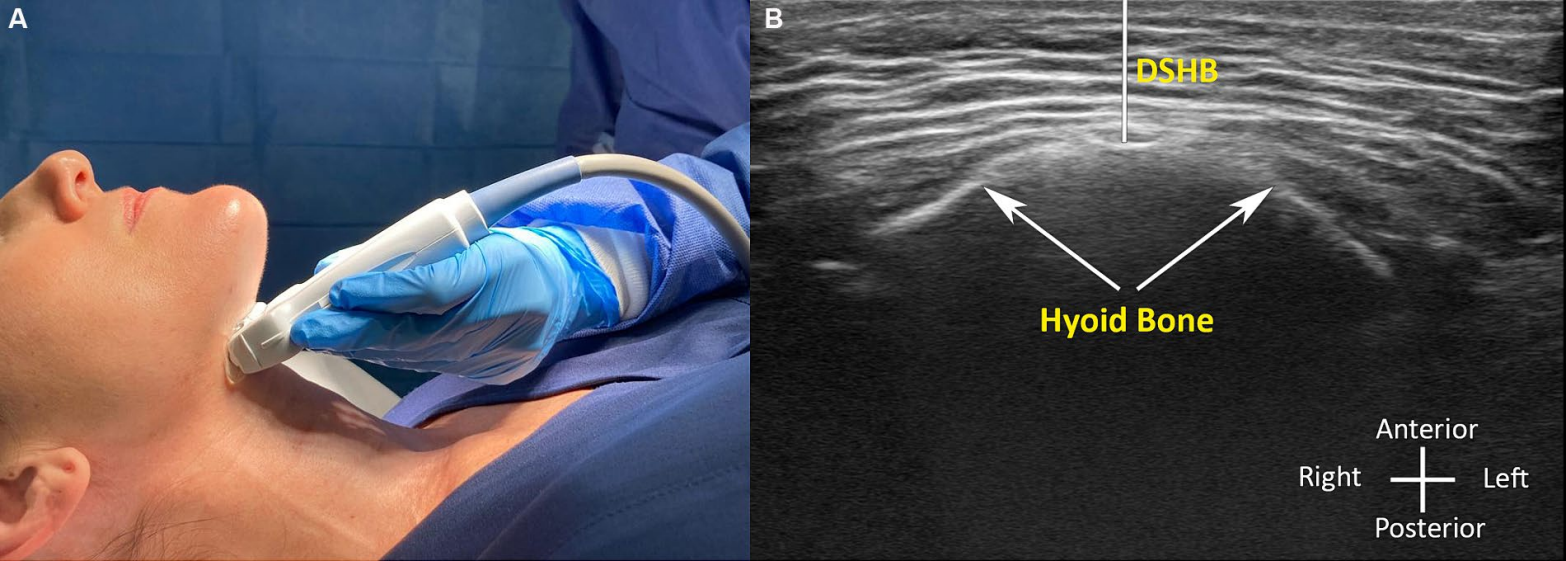
**(A,B)** Ultrasound image measurements in awake patient with the corresponding probe position. **(B)** Distance from skin to the hyoid bone (DSHB).

**Figure 2:**
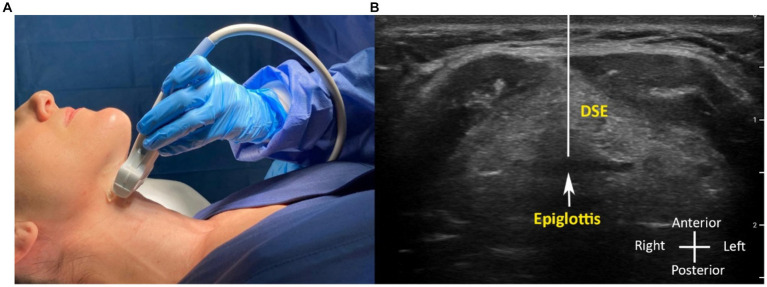
**(A,B)** Ultrasound image measurements in awake patient with the corresponding probe position. **(B)** Distance from skin to the epiglottis (DSE).

**Figure 3:**
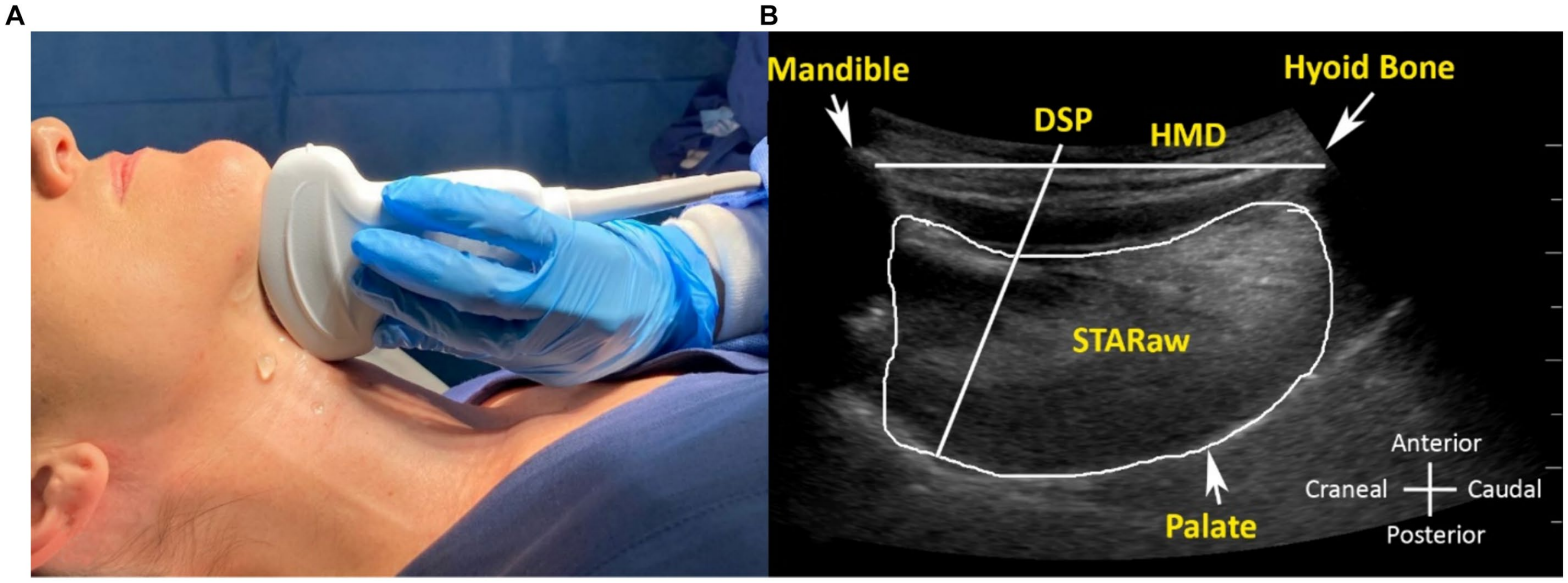
**(A,B)** Ultrasound image measurements in awake patient with the corresponding probe position. **(B)** Hyomental distance (HMD), sagittal tongue area in awake patient (STARaw) and distance from skin to the deepest part of palate (DSP).

Prior to general anesthesia induction, the following five ultrasound measurements were performed in the awake patient and in neutral head position: (1) the distance from the skin to the hyoid bone (DSHB), (2) the distance from the skin to the epiglottis (DSE), (3) the hyomental distance in the neutral position (HMDn), (4) the sagittal tongue area in awake patients (STARaw), and (5) the distance from the skin to the deepest part of the palate (DSP). Additionally, hyomental distance (HMDe) was measured in the head-extended position. The hyomental distance ratio (HMDr) was calculated by this formula: HMDe/HMDn. To facilitate the reproducibility of this pilot study, all the procedures and protocols needed for an adequate airway sonography assessment are described in Figures 1–3.

### General anesthesia induction and tracheal intubation

Standard non-invasive anesthesia monitoring (non-invasive blood pressure, electrocardiogram, pulse oximetry, capnography, neuromuscular monitoring and hypnotic depth) was applied in all patients for continuous surveillance during anesthesia. Proper preoxygenation was confirmed by an E_T_O_2_ greater than 90%, and general anesthesia was induced with propofol, fentanyl and rocuronium. A first attempt of direct laryngoscopy with a Macintosh blade (Riester, Jungingen, Germany) was used to assess C-L grades. After that, a second laryngoscopy was performed using a McGrath™ Mac videolaryngoscope (Aircraft Medical, Edinburgh, United Kingdom) to obtain an indirect laryngoscopy vision. A size 3 Macintosh blade for females or a size 4 for males were used during laryngoscopies. The POGO score was employed to describe the videolaryngoscopic view because of its good intra-and interobserver precision and consistency (15, 16). Finally, tracheal tube insertion maneuver was performed.

Tracheal intubation using videolaryngoscopy was performed by anesthesiologists who had a minimum of 2 years of clinical experience and/or who had performed at least 100 intubations with the McGrath™ Mac videolaryngoscope.

We adhered to the instructions outlined by the McGrath™ Mac videolaryngoscope manufacturer for the intubation methodology.^1^

To evaluate the difficulty of tracheal intubation using videolaryngoscopy, the following recently introduced simplified score was used (17).

a. GRADE 0—Easy tracheal intubation using videolaryngoscopy (E-VL): Successful attempts (first pass success at tracheal intubation) without any adjunct needed were achieved using the McGrath™ Mac videolaryngoscope.
b. GRADE 1—Difficult tracheal intubation using videolaryngoscopy (D-VL): Successful attempts were achieved with an adjunct such as a malleable stylet, a Frova intubating catheter (Cook Medical, Bjæverskov, Denmark) or a second hyperangulated blade or videolaryngoscope (18). Flexible bronchoscopy was ready to use as a rescue strategy if more than two unsuccessful intubation attempts occurred.

During ultrasonography and videolaryngoscopy performance in the anesthetized patient, respiratory and hemodynamic surveillance was provided to ensure patient safety. Complications were defined by desaturation (SatO_2_ < 92%), esophageal intubation or dental trauma (8). Furthermore, adequate hypnotic depth monitorization (BIS below 50) (Covidien, Mansfield, United States) and a correct neuromuscular relaxation (TOF = 0) (General Electric, GE Medical Systems, Jiangsu, China) were also provided.

### Post-induction ultrasound measurements

**Figure 4:**
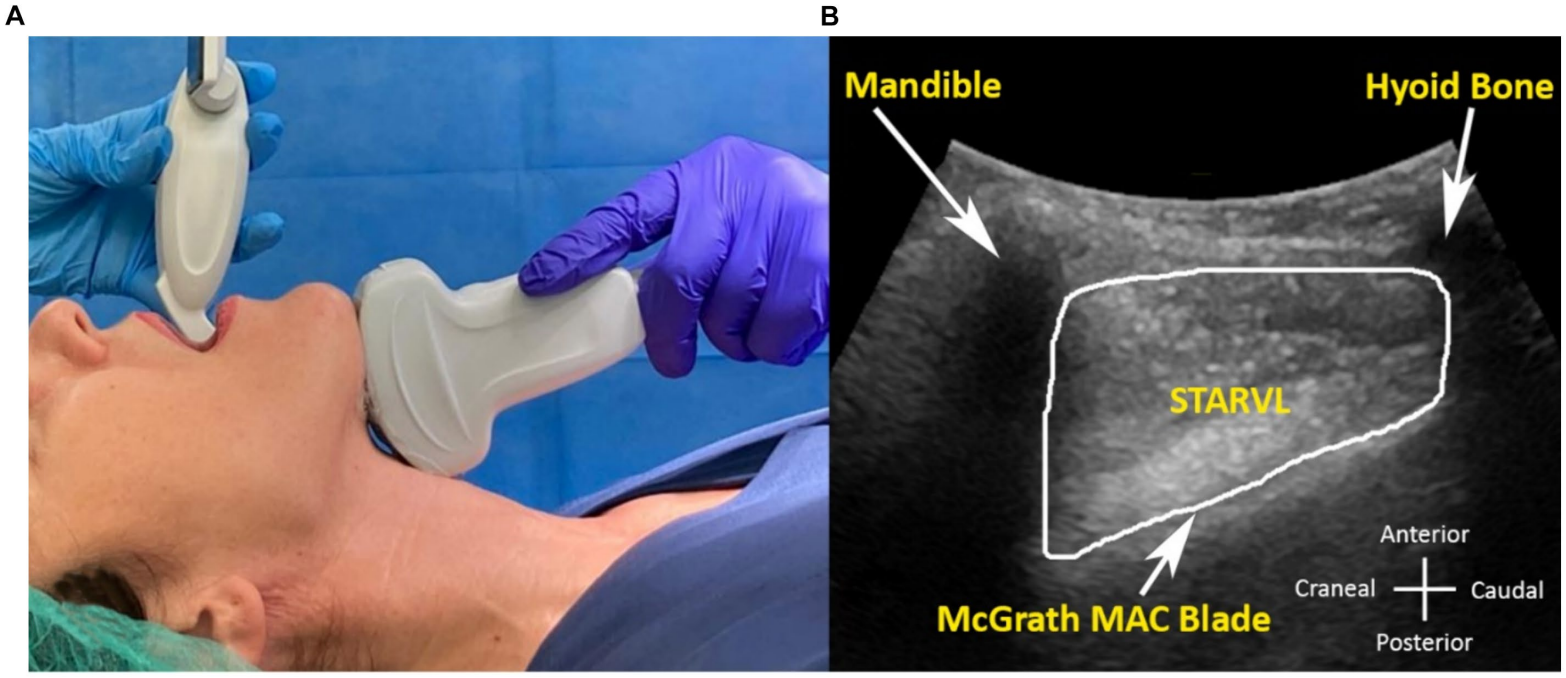
**(A,B)** Ultrasound image measurements in the anesthetized patient during videolaryngoscopy with McGrath^™^ Mac blade and the corresponding probe position. **(B)** Sagittal tongue area compressed by the videolaryngoscope (STARVL).

One ultrasound measurement was taken during videolaryngoscopy, with the patient anesthetized and with the head in the “sniffing” position, as recommended by the manufacturer. This parameter was the sagittal tongue area compressed by the videolaryngoscope (STARVL). In this scenario, one operator performed the videolaryngoscopy, while another conducted ultrasound examination for patient safety (Figure 4).

### Outcomes

The primary outcome was to evaluate the efficacy of clinical parameters and ultrasound measurements to predict tracheal intubation difficulty using a McGrath™ Mac blade videolaryngoscope, in adult patients undergoing general anesthesia for elective surgical procedures.

The secondary outcome was to evaluate the association between the POGO score and the difficulty of tracheal intubation using videolaryngoscopy (regarding a “can see, cannot intubate” scenario).

A prospective observational study design was employed to assess the feasibility of our study objectives for future international multicenter collaborative research. The present study protocol involved recruiting patients within a designated time period and performing interventions according to predetermined criteria (19).

### Statistical analysis

Ultrasound measurements should predict difficulty in at least 85% of the intubations for direct laryngoscopy (9, 10), but this percentage has not been calculated for videolaryngoscopy. As the incidence of difficult intubations is about 5 to 10% of all intubations (18), it would be necessary to study at least 112 patients to obtain statistical significance, accepting an alpha error of 0.05. However, given the pilot nature of this study, the determination of a specific sample size was deemed not mandatory.

Univariate analysis was used to examine the correlation between demographic, clinical, and ultrasound variables in relation to difficult tracheal intubation using videolaryngoscopy. Qualitative variables were subjected to Chi-square or Fisher’s exact tests, while quantitative variables were evaluated using Student’s *t*-test or Wilcoxon’s non-parametric test. A logistic regression model was used to analyze the association of each variable with difficult tracheal intubation using videolaryngoscopy (20). The ROC curve methodology was used for variable categorization, and the optimal cut-off point was identified as the value that maximizes the sum of sensitivity and specificity (21).

A multivariate analysis was performed using a logistic regression model (Spearman’s rank correlation coefficient). Model calibration was performed by the application of the Hosmer and Lemeshow goodness-of-fit test.

For the secondary outcome and to establish the relationship and concordance among variables, Spearman’s comparison coefficient and Cohen’s Kappa Index (ranging from −1 to +1) were used. This is a statistic index that is used to measure inter-rater reliability (and also intra-rater reliability) for qualitative (categorical) items. A *p*-value <0.05 was considered statistically significant. Data processing and statistical analysis were performed using SPSS software version 25.0 (IBM, Armonk, New York).

The accuracy, sensitivity, specificity, positive predictive value (PPV) and negative predictive value (NPV) of the indicators were calculated with the Epidat 3.1 program (SERGAS, Galicia, Spain).

Considering the pilot nature of our study, feasibility is contingent upon several criteria: the ability to recruit an adequate number of patients within a short timeframe, absence of intervention-related complications, and identification of any ultrasound measurements correlated with difficult airway management.

## Results

### Demographic and clinical characteristics

**Figure 5:**
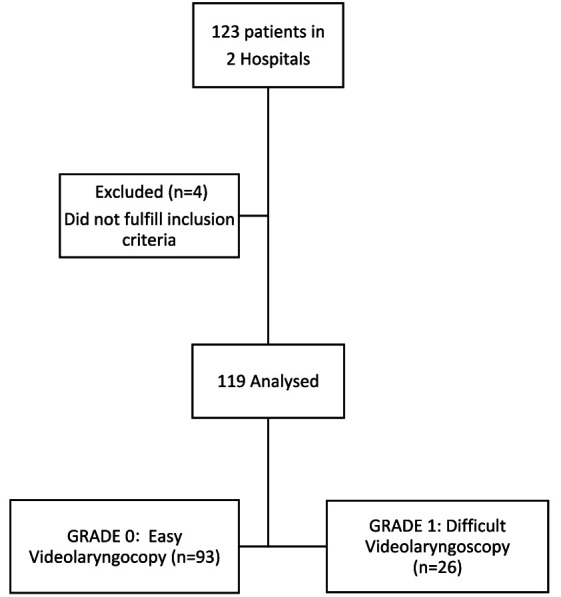
Patient flow diagram.

A total of 119 patients from two hospitals were enrolled in the study (Figure 5). Patient characteristics are shown in Table 1. Ninety-three patients (78.2%) were successfully intubated using the McGrath™ Mac videolaryngoscope, with no additional adjuvants (GRADE 0: E-VL, as defined in the methods section), while 26 patients (21.8%) experienced difficult tracheal intubation using videolaryngoscopy (GRADE 1: D-VL). The subgroup of patients with D-VL, required various adjuncts, including a stylet (50%), a Frova catheter (19.2%) or a hyperangulated blade (30.8%) (Airtraq^®^ -Prodol Meditec, Vizcaya, Spain, or McGrath™ X Blade-Aircraft Medical, Edinburgh, United Kingdom). No patient required awakening or rescue strategy. No major complications or adverse events occurred during the intubation process.

**Table 1:** Patient characteristics, clinical screening tests, and ultrasound measurements.

Parameter	Easy videolaryngoscopy *n* = 93	Difficult videolaryngoscopy *n* = 26	*p*-value
Patient characteristics			
Gender (male/female) n (%)			0.053
Male	56 (60%)	21 (81%)	
Female	37 (40%)	5 (19%)	
Age, (years), mean ± [SD]	57.7 ± [16.3]	60.6 ± [12.4]	0.405
BMI, (kg.m^−2^), mean ± [SD]	25.3 ± [3.5]	28.9 ± [3.3]	<0.001
ASA physical status, n (%)			0.418
I	14 (15%)	2 (8%)	
II	71 (76%)	20 (77%)	
III	8 (9%)	4 (15%)	
Clinical screening tests			
Modified Mallampati score, n (%)			0.002
I-II	84 (90%)	17 (65%)	
III-V	9 (10%)	9 (35%)	
Thyromental distance (cm), mean ± [SD]	7.6 ± [0.9]	7.3 ± [1]	0.084
Sternomental distance (cm), mean ± [SD]	13.9 ± [1.6]	13.4 ± [1.5]	0.14
Interincisor distance (cm), mean ± [SD]	4.2 ± [0.7]	4 ± [0.9]	0.28
Upper lip bite test, n (%)			0.87
I-II	90 (97%)	25 (96%)	
III	3 (3%)	1 (4%)	
Neck circumference (cm), mean ± [SD]	38.8 ± [4.2]	43 ± [3.6]	<0.001
Ultrasound measurements performed in awake patient			
Distance skin to hyoid bone (cm), mean ± [SD]	0.99 ± [0.25]	1.36 ± [0.29]	<0.001
Distance skin to epiglottis (cm), mean ± [SD]	2.00 ± [0.33]	2.56 ± [0.39]	<0.001
Hyomental distance ratio, mean ± [SD]	1.13 ± [0.06]	1.04 ± [0.06]	<0.021
Distance skin to deepest part of palate (cm), mean ± [SD]	5.6 ± [0.56]	6.40 ± [0.50]	<0.001
Sagittal tongue area (cm^2^) (STARaw), mean ± [SD]	20.27 ± [2.53]	26.44 ± [2.88]	<0.001
Ultrasound measurements under general anesthesia during videolaryngoscopy			
Sagittal tongue area (cm^2^) (STARVL), mean ± [SD]	11.84 ± [1.69]	17.19 ± [1.79]	<0.001

### Association between patient characteristics, clinical parameters, ultrasound parameters, and tracheal intubation difficulty

There was a significant difference in BMI between individuals with difficult and no difficult intubation, but no differences were found in sex, age, or ASA status. Regarding clinical parameters, only MMS and NC were statistically significant. Concordance analysis between the C-L grade evaluated by direct laryngoscopy (as a predictor of difficulty) and D-VL, yielded a Cohen’s Kappa of 0.815, indicating a nearly perfect agreement (95% of the patients with C-L grades 3 and 4 were classified as D-VL).

Finally, all the ultrasound parameters showed statistically significant differences in relation to D-VL. The patients´ characteristics, clinical airway assessment data, and ultrasound measurements are described in Table 1.

### Roc analysis in predicting difficult intubation

**Figure 6:**
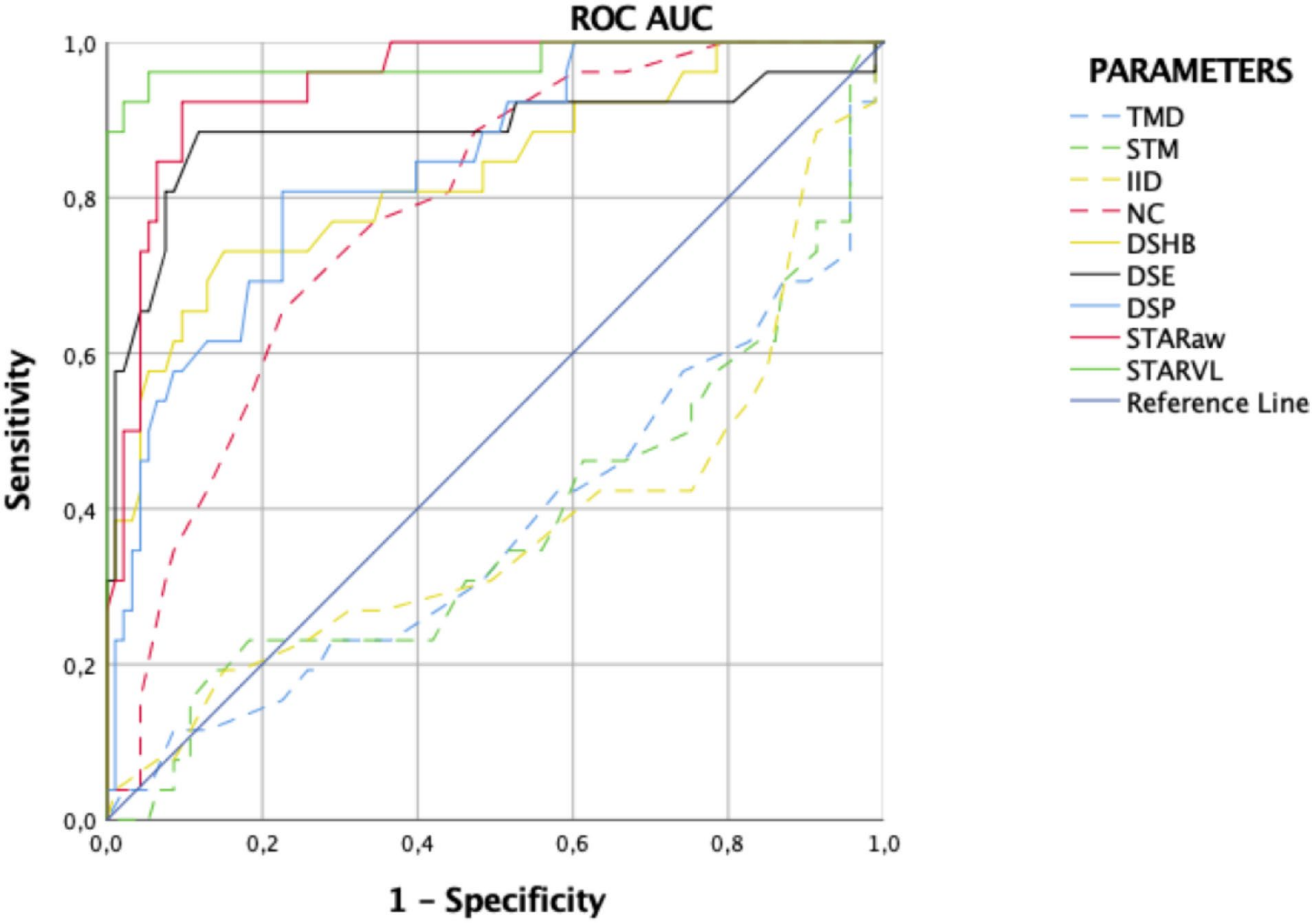
Receiver operating characteristic (ROC) for clinical tests and ultrasound measurements for difficult intubation with videolaryngoscope. Clinical parameters (dotted lines): thyromental distance (TMD; blue dotted line), sternomental distance (SMD; green dotted line), interincisal distance (IID; yellow dotted line) and neck circumference (NC; red dotted line). Ultrasound measurements taken in awake patients (solid lines): distance from skin to the hyoid bone (DSHB; yellow solid line), distance from skin to the epiglottis (DSE; black solid line), sagittal tongue area in awake patients (STARaw; red solid line), and distance from skin to the deepest part of palate (DSP; blue solid line). Ultrasound measurement in anesthetized patients (solid lines): compressed sagittal tongue area (STARVL; green solid line).

ROC curve analysis revealed the superior performance of ultrasound parameters over clinical variables in identifying D-VL. NC emerged as the only clinical variable with significant discriminatory capacity, as depicted in Table 1 and Figure 6. The calculated Youden index, sensitivity, specificity, PPV, and NPV for all variables, along with the respective cut-off points for ultrasound measurements, are summarized in Table 2.

**Table 2:** Diagnostic accuracy of clinical and ultrasound parameters, cut-off points for ultrasound variables, and logistic regression model for predicting difficult intubation using videolaryngoscopy.

Parameter	AUC (95%CI)	Cut-off points	Se	Sp	PPV	NPV	Y-I	*p*-value
Clinical screening tests								
Thyromental distance (TMD)	0.37 (0.24–0.51)		30.77	90.32	47.06	82.35	0.21	0.054
Sternomental distance (SMD)	0.38 (0.25–0.51)		23.08	92.47	46.15	81.13	0.16	0.061
Interincisor distance (IID)	0.37 (0.24–0.51)		42.31	84.95	44.00	84.04	0.27	0.057
Neck circumference (NC)	0.78 (0.69–0.87)		65.38	77.42	44.74	88.89	0.43	<0.001
Ultrasound measurements performed in awake patient								
Distance skin hyoid bone (DSHB)	0.83 (0.73–0.92)	1.15 cm	76.92	70.97	42.55	91.67	0.48	<0.001
Distance skin epiglottis (DSE)	0.89 (0.78–0.98)	2.45 cm	88.46	88.17	67.65	96.47	0.77	<0.001
Distance skin to deepest part of palate (DSP)	0.84 (0.75–0.92)	6.00 cm	80.77	77.42	50.00	93.51	0.58	<0.001
Sagittal tongue area (STARaw)	0.95 (0.91–0.99)	23.33 cm^2^	92.31	90.32	72.73	97.67	0.83	<0.001
Ultrasound measurements under general anesthesia during videolaryngoscopy								
Sagittal tongue area (STARVL)	0.98 (0.93–1.00)	14.41 cm^2^	96.15	94.62	83.33	98.88	0.91	<0.001
Distance skin to epiglottis (DSE) + Distance skin to deepest part palate (DSP) + Sagittal tongue area (STARaw)								
Ultrasound model	0.95 (0.91–0.99)		92.31	94.62	82.76	97.78	0.87	<0.001

VL, videolaryngoscope; AUC, area under the curve (CI, confidence interval); Se, sensitivity; Sp, specificity; PPV, predictive positive value; NPV, negative predictive value; Y-I, Youden’s Index.

In awake patients, the STARaw (sagittal tongue area in awake patients) demonstrated the most favorable performance, with a cut-off point of 23.3 cm^2^. On the other hand, in anesthetized patients this parameter was the STARVL, with a cut-off point of 14.4 cm^2^.

### Multivariate logistic regression analysis

A multivariate logistic regression analysis which integrated clinical and preoperative ultrasound parameters found that STARaw, DSE (distance from the skin to the epiglottis), and DSP (distance from the skin to the deepest part of the palate) were strongly associated with D-VL. STARaw was the most influential variable (*p* < 0.001), with an odds ratio (OR) of 23.76 (95% CI 3.95–142.93), while DSE also displayed significant association with an OR of 10.25 (95% CI 1.91–54.93). The comprehensive analysis including STARaw, DSE, and DSP obtained the most favorable results, surpassing the evaluations of individual variables. This model achieved a ROC AUC of 0.952 (95% CI 0.91–0.99), with a sensitivity of 92.3%, specificity of 94.5%, PPV of 82.76%, NPV of 97.78%, and a Youden Index of 0.87, as detailed in the last row of Table 2.

### Secondary outcome

Concordance analysis between POGO score and D-VL, showed a negative Cohen’s Kappa of −0.34. These data suggest difficulty in tracheal intubation despite an adequate glottic view (“can see, cannot intubate” scenario). These results are depicted in Table 3.

**Table 3:** Secondary outcome.

Parameter	Easy videolaryngoscopy *n* = 93	Difficult videolaryngoscopy *n* = 26	Kappa index	*p*-value
POGO* (%), n (%)			−0.34	<0.001
25%	1 (1%)	5 (19%)		
50%	6 (6%)	11 (42%)		
75%	27 (30%)	6 (23%)		
100%	59 (63%)	4 (16%)		

*POGO, percentage of glottic opening.

During the study we were able to recruit the targeted number of patients within the specified timeframe, with no reported complications associated with the intervention. Furthermore, analysis of ultrasound measurements revealed notable correlations with indicators of difficult airways, thereby supporting the feasibility of our study approach.

Finally, it is important to note that no major complications nor adverse events occurred during the intubation process in any of the patients, as shown in Table 4.

**Table 4:** Intubation process variables.

	Easy videolaryngoscopy *n* = 93	Difficult videolaryngoscopy *n* = 26	*p*-value
Complications			
Desaturation (SpO_2_ < 92%)	0 (0.0%)	1 (3.8%)	0.218
Esophageal intubation	0 (0.0%)	0 (0.0%)	NSS
Dental trauma	0 (0.0%)	0 (0.0%)	NSS
C-L grade n (1–2/3–4)			
	92/1	6/25	<0.001
POGO* (%) n (25/50/75/100)			
	1/6/27/59	5/11/6/4	<0.001

*POGO, percentage of glottic opening.

## Discussion

Our study in non-obese patients highlights the potential of three specific airway ultrasound measurements to predict difficulty in tracheal intubation using the McGrath™ Mac videolaryngoscope. This predictive model includes the sagittal tongue area in awake patients (STARaw), the distance from the skin to the epiglottis (DSE), and the distance from the skin to deepest part of the palate (DSP).

Previous investigations have extensively explored the utility of ultrasound to assess the probability of encountering difficulties with direct laryngoscopy (9–12). However, our study represents a pioneering investigation in the relationship between ultrasound findings and challenging videolaryngoscopy.

In our research, several established risk factors (13) by the Canadian Airway Focus group for difficult tracheal intubation using videolaryngoscopy were confirmed. Those factors included male sex, increased NC, high C-L grade, and enlarged tongue size. On the contrary, we did not observe this correlation with TMD, STM, IID, and ULBT. Moreover, our study identified positive associations between high BMI and MMS with challenging intubation using videolaryngoscopy, these findings have not been previously described specifically for the McGrath™ Macintosh blade videolaryngoscope.

Macroglossia has been described as a predictor of difficulty in tracheal intubation using videolaryngoscopy (13) and direct laryngoscopy (22, 23). However, to date, no specific cut-off values have been published for tongue size quantified by ultrasonography. The size and compressibility of the tongue have emerged as especially relevant factors in our study. STARaw and STARVL showed consistent correlations with difficult videolaryngoscopy, each of them with clear cut-off points (23.3 and 14.4 cm^2^, respectively). These findings suggest that tongue characteristics may pose significant challenges during tracheal intubation using videolaryngoscopy, which highlights the importance of assessing tongue size in airway evaluation.

For DSHB and DSE, cut-off points of 1.15 and 2.45 cm were determined, respectively. These values are closely aligned with those previously identified for difficult direct laryngoscopy in a previous study from our research group (24, 25), and other authors in a similar population (26). The higher the values are, the greater is the difficulty. Therefore, these ultrasound parameters appear to be versatile and useful for discerning difficulties associated with tracheal intubation with a Macintosh-type blade, both in direct laryngoscopy and videolaryngoscopy.

Atlanto-occipital and atlanto-axial movements limitations, described as HMDr by ultrasonography, have been also previously published in difficult direct laryngoscopy studies (27, 28). The results of our research also showed an increase in the difficulty of tracheal intubation using videolaryngoscopy in relation to HMDr. As HDMr is a ratio, lower HDMr values indicate a higher difficult tracheal intubation probability due to these neck movements limitations.

To our knowledge, the distance from the skin to the deepest part of the palate (DSP) measurement has not been previously published. In our study, DSP is also correlated with D-VL. The greater this distance, the larger the patient’s oral cavity is and therefore the better you can maneuver with both the videolaryngoscope and the endotracheal tube.

Finally, our multivariate logistic regression analysis revealed that the combination of STARaw, DSE and DSP provided the most accurate multiparametric ultrasound model for detecting difficulties during videolaryngoscopic intubation. In clinical practice, videolaryngoscopy is increasingly replacing direct laryngoscopy for tracheal intubation. This trend is evident in airway management guidelines updated by the ASA (29) or the Canadian Airway Focus Group (13, 18) and supported by other authors (30, 31). The integration of POCUS into routine clinical practice offers a safe, portable, and accessible means of assessing airway anatomy in real-time (5, 6). The combination of ultrasound with videolaryngoscopy could enhance decision-making strategies during airway management and prove accurate data which could determine different choices in clinical practice.

For the secondary outcome, our study confirmed the association between POGO score and difficulty in intubation, indicating the presence of the “you-see that you-fail” phenomenon (32) with videolaryngoscopy (“can see, cannot intubate” scenario”) (33). It could be related with the fact that the angle of view with a videolaryngoscope is 4 times that of a classic laryngoscope, which achieves a much better visualization of the airway (34).

Limitations of our study include the inability to blind airway management and ultrasound measurements. Furthermore, this is a non-randomized design which included only a specific European population and one type of videolaryngoscope, which may limit generalizability. These findings cannot be extrapolated to other videolaryngoscopes, especially those with hyperangulated or channeled blades.

Ultrasonographic procedures were standardized and conducted by experienced airway ultrasonopraphers. However, airway POCUS performance has a short learning curve (6). In this paper, several ultrasonography figures, measurements, and probe positioning are explained to facilitate reproducibility.

The C-L grade was recorded using a Macintosh blade for direct laryngoscopy before performing a second indirect laryngoscopy with the McGrath videolaryngoscope. Theoretically, this maneuver might have the potential to increase edema and/or trauma. For this reason, in the next study to be carried out, the protocol will be changed and direct and indirect laryngoscopy will be performed with the same device, using McGrath videolaryngoscope. To do this, the camera will be covered during direct laryngoscopy and uncovered for indirect laryngoscopy.

Another limitation could be the requirement of a second operator during videolaryngoscopy to measure STARVL.

Finally, we decided not to use a stylet for intubation because several studies performed with a Macintosh blade have shown that there are no advantages to its use in patients with easy airways (35). Also, its application could produce potential complications (36, 37), or it was suggested as a limitation (7).

The strengths of our study include the detection of possible challenging videolaryngoscopy using a Macintosh blade by prior ultrasound assessment. Anticipation and decision making could be facilitated if difficulties are foreseen. In such airway management, the use of an adjuvant (stylet or FROVA) or a hyperangulated blade could be chosen for the first attempt.

## Conclusion

Our study suggests that there are promising reliable ultrasound measurements which could predict difficult tracheal intubation using videolaryngoscopy, specifically the sagittal tongue area in awake patients (STARaw), the distance from the skin to the epiglottis (DSE) and the distance from the skin to the palate (DSP). These findings provide valuable information to optimize airway management strategies, especially in the selection of appropriate techniques and equipment for a first attempt and safe intubation.

## Data availability statement

The original contributions presented in the study are included in the article/supplementary material, further inquiries can be directed to the corresponding author.

## Ethics statement

The studies involving humans were approved by the Ethical Committees of Navarra University Hospital (Pamplona, Spain) and Euskadi (Vitoria, Spain). The studies were conducted in accordance with the local legislation and institutional requirements. Written informed consent for participation in this study was provided by the participants’ legal guardians/next of kin. Written informed consent was obtained from the individual(s), and minor(s)’ legal guardian/next of kin, for the publication of any potentially identifiable images or data included in this article.

## Author contributions

MF-V: Conceptualization, Formal analysis, Investigation, Methodology, Project administration, Validation, Writing – original draft, Writing – review & editing, Data curation. NL-C: Conceptualization, Funding acquisition, Investigation, Methodology, Writing – original draft, Writing – review & editing, Data curation. MG-A: Writing – review & editing. JÁ-A: Writing – review & editing. MV-S: Writing – review & editing. RG: Supervision, Writing – review & editing. EM-H: Writing – review & editing, Conceptualization, Visualization. DL-L: Conceptualization, Methodology, Supervision, Validation, Writing – original draft, Writing – review & editing.

## Footnotes

^1^https://www.medtronic.com/covidien/en-us/support/products/intubation/mcgrath-mac-videolaryngoscope.html
